# A 12-year record of intertidal barnacle recruitment in Atlantic Canada (2005–2016): relationships with sea surface temperature and phytoplankton abundance

**DOI:** 10.7717/peerj.2623

**Published:** 2016-10-25

**Authors:** Ricardo A. Scrosati, Julius A. Ellrich

**Affiliations:** Department of Biology, St. Francis Xavier University, Antigonish, Nova Scotia, Canada

**Keywords:** Intertidal, Barnacle, Recruitment

## Abstract

On the Gulf of St. Lawrence coast of Nova Scotia (Canada), recruitment of the barnacle *Semibalanus balanoides* occurs in May and June. Every year in June between 2005 and 2016, we recorded recruit density for this barnacle at the same wave-exposed rocky intertidal location on this coast. During these 12 years, mean recruit density was lowest in 2015 (198 recruits dm^−2^) and highest in 2007 (969 recruits dm^−2^). The highest recruit density observed in a single quadrat was 1,457 recruits dm^−2^ (in 2011) and the lowest was 34 recruits dm^−2^ (in 2015). Most barnacle recruits appear during May, which suggests that most pelagic larvae (which develop over 5–6 weeks before benthic settlement) are in the water column in April. An AICc-based model selection approach identified sea surface temperature (SST) in April and the abundance of phytoplankton (food for barnacle larvae, measured as chlorophyll-*a* concentration –Chl-*a*–) in April as good explanatory variables. Together, April SST and April Chl-*a* explained 51% of the observed interannual variation in recruit density, with an overall positive influence. April SST was positively related to March–April air temperature (AT). April Chl-*a* was negatively related to the April ratio between the number of days with onshore winds (which blow from phytoplankton-limited offshore waters) and the number of days with alongshore winds (phytoplankton is more abundant on coastal waters). Therefore, this study suggests that climatic processes affecting April SST and April Chl-*a* indirectly influence intertidal barnacle recruitment by influencing larval performance.

## Introduction

Recruitment is a key demographic step that replenishes populations and ensures their persistence, so it has received considerable attention in ecology (Caley et al., 1996; Beck et al., 2001; Palumbi & Pinsky, 2014). Because of their ease for monitoring, barnacles have become important model organisms to study recruitment. For barnacles, recruitment refers to the appearance of new benthic organisms that have metamorphosed after pelagic cyprid larvae have settled on the substrate (Ellrich et al., 2016). Barnacles are often abundant in rocky intertidal habitats, which are therefore places where barnacle recruitment has been mostly studied (Jenkins et al., 2000; Navarrete, Broitman & Menge, 2008; Lathlean, Ayre & Minchinton, 2013; Menge et al., 2015; Barbosa et al., 2016).

As the complexity of coastal systems cannot be replicated in the laboratory, barnacle recruitment and its external drivers have been investigated mainly through mensurative field studies. Particularly useful are long-term records of recruitment coupled with environmental data (Kendall et al., 1985; Menge & Menge, 2013). Long-term records of intertidal barnacle recruitment exist for the NE Pacific coast (28 years by Menge et al., 2011; B.A. Menge, 2016, personal communication), the SE Pacific coast (nine years by Navarrete, Broitman & Menge, 2008), and the NE Atlantic coast (13 years by Kendall et al., 1985, and 30 years by Abernot-Le Gac et al., 2013), which harbour temperate biotas. For the NW Atlantic coast, another temperate coastal system, intertidal barnacle recruitment has been documented (Bertness, 1989; Minchinton & Scheibling, 1991; Petraitis & Vidargas, 2006; Bertness, Gaines & Wahle, 1996; Leonard, Ewanchuk & Bertness, 1999; Leonard, 2000; Pineda, Riebensahm & Medeiros-Bergen, 2002; Cole et al., 2011; Ellrich, Scrosati & Molis, 2015), but long-term records of at least a decade are unavailable. In fact, no such long-term recruitment dataset seems to exist for any rocky intertidal invertebrate from this coast.

To address this knowledge gap, in 2005 we started a monitoring program to record intertidal barnacle recruitment every year at the same location in Atlantic Canada. This paper reports the results until 2016. Our first objective is to document the interannual changes in barnacle recruitment during this 12-year period. Sea surface temperature (SST) and coastal phytoplankton abundance are often important drivers of intertidal barnacle recruitment (Menge & Menge, 2013; Mazzuco et al., 2015). While water temperature universally affects the performance of aquatic ectotherms (Payne et al., 2016; Seabra et al., 2016), phytoplankton feeds the pelagic nauplius larvae (the stages preceding the cyprid stage) and recruits of barnacles, as they are filter-feeders (Singarajah, Moyse & Knight-Jones, 1967; Anderson, 1994; Jarrett, 2003; Gyory, Pineda & Solow, 2013). Therefore, our second objective is to evaluate how barnacle recruitment was related to SST and phytoplankton abundance during our study period in search of signals of external forcing in this system.

## Materials and Methods

**Figure 1:**
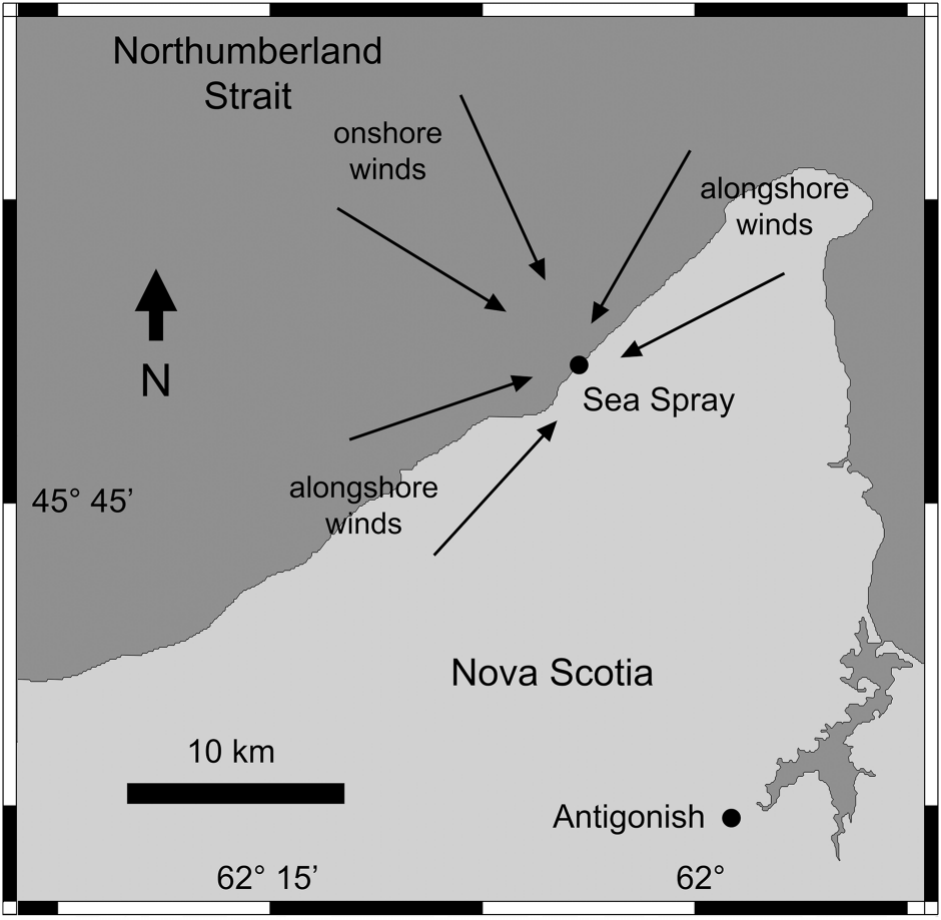
Map indicating the position of the surveyed location (Sea Spray) on the southern coast of the Gulf of St. Lawrence, Nova Scotia, Canada. The arrows indicate common onshore winds and alongshore winds.

**Figure 2:**
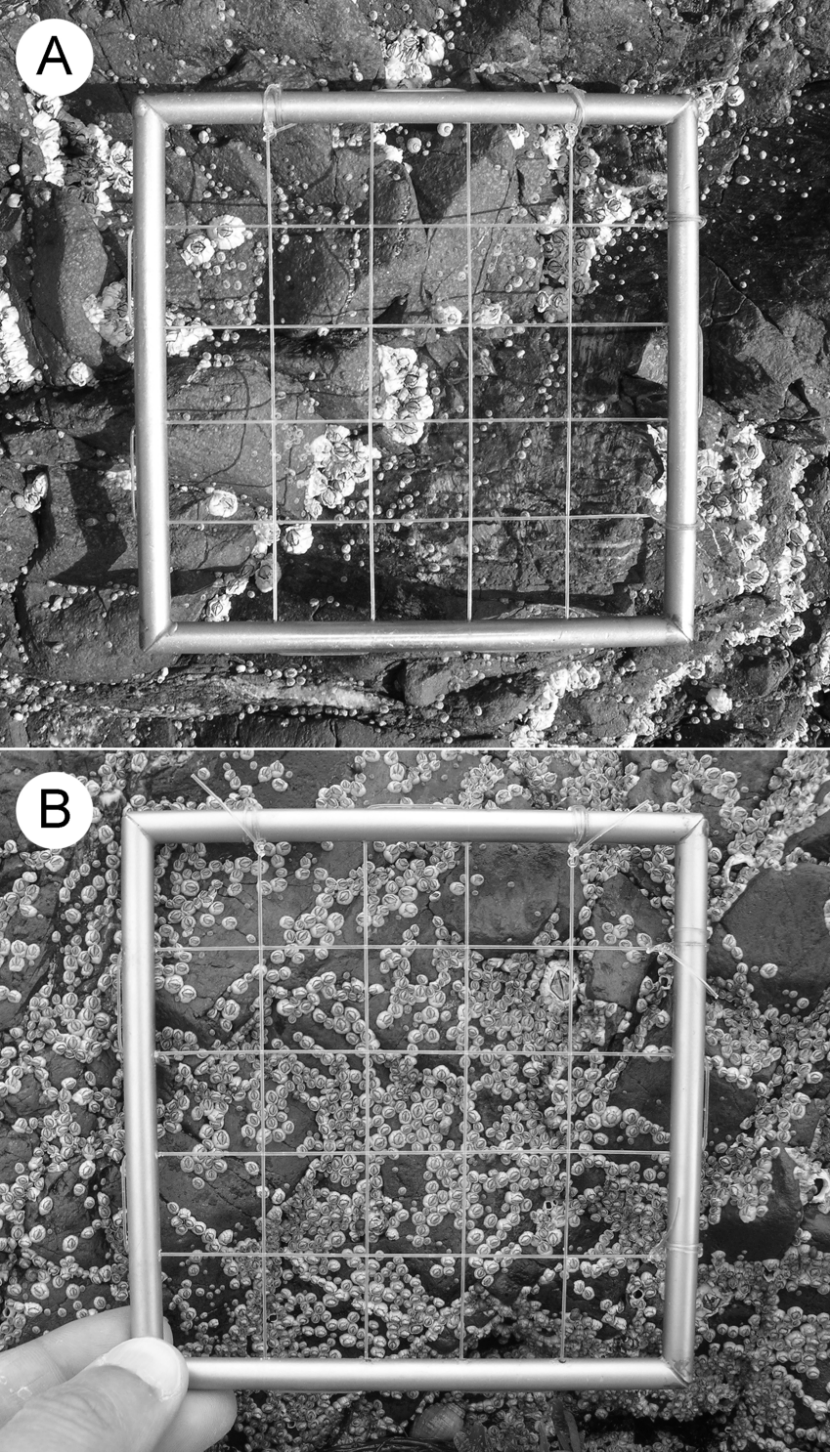
Pictures of the studied habitat taken in June in different years. (A) View showing several adult barnacles that survived the previous winter and many recruits that appeared during the spring. (B) View showing a higher abundance of recruits and only one adult barnacle.

The study location is Sea Spray (45°46.38′N, 62°8.67′W), located near Arisaig, on the southern coast of the Gulf of St. Lawrence, Nova Scotia, Canada (Fig. 1). The surveyed intertidal habitats are wave-exposed, as they face open waters without any physical obstruction. In-situ measures of daily maximum water velocity taken during the summer and fall of 2005 ranged between 4–8 m s^−1^ (Scrosati & Heaven, 2007). The intertidal substrate of the surveyed habitats is stable volcanic bedrock with a moderate slope and rugosity (Fig. 2).

On this coast, *Semibalanus balanoides* is the only intertidal barnacle species (Scrosati & Heaven, 2007). It is a cross-fertilizing hermaphrodite that, in Atlantic Canada, mates in autumn, breeds in winter, and releases pelagic larvae in spring (Bousfield, 1954; Crisp, 1968). The sea surface in this region freezes in winter (Galbraith et al., 2015). After the ice melts in late winter or early spring, barnacle recruits appear on the substrate during a limited recruitment season between early May and mid- to late June (Ellrich, Scrosati & Molis, 2015). Recruits appear throughout the full vertical intertidal range, which is 1.8 m on this coast (MacPherson & Scrosati, 2008). For this study, we measured barnacle recruitment at an elevation of 2/3 of the intertidal range. Shortly before the 2005 recruitment season, we determined the position of a permanent transect line by establishing stainless steel nails on the substrate at 1.2 m of elevation above chart datum (lowest normal tide, or 0 m). Then, at the second or third week of June every year from 2005 to 2016 (Table 1), we measured the density of barnacle recruits in 29–33 (Table 1) 10 × 10 cm quadrats randomly positioned right above the transect line. Each quadrat was photographed to enable accurate recruit counting on a computer. Because of intense ice scour in winter (Scrosati & Heaven, 2006), the surveyed intertidal habitats in spring consist of mostly bare rock, offering abundant space for barnacle recruitment (Fig. 2). Other macroscopic organisms occurring in such habitats in early spring are mostly only a few adult barnacles from previous years (MacPherson, Scrosati & Chareka, 2008). Fucoid algae and snails are rare in these habitats at that time of the year. Barnacle recruits are easily identified because of their small size (1–2 mm in basal diameter) compared with adult barnacles (Fig. 2).

**Table 1:** Dates in which barnacle recruit density was measured and sample size (number of quadrats) used for each date.

Measurement date	Sample size
8 June 2005	30
7–10 June 2006	30
14 June 2007	30
6 June 2008	29
10 June 2009	32
12 June 2010	31
6 June 2011	33
8 June 2012	33
6 June 2013	33
15 June 2014	32
15 June 2015	32
18 June 2016	32

We obtained data on SST and chlorophyll-*a* concentration (Chl-*a*, proxy for phytoplankton abundance) from the OceanColor Web database from the National Aeronautics and Space Administration using SeaDAS software (National Aeronautics and Space Administration, 2016). This website provides data measured by three satellites that were operational at different times during our study period: MODIS-Aqua measured SST between 2005–2011, MERIS measured Chl-*a* between 2005–2011, and VIIRS measured SST and Chl-*a* between 2012–2016. We calculated monthly means of SST and Chl-*a* using the data for the 4 × 4 km cell that contains Sea Spray. When no data were available for that cell (April SST in 2006 and 2008, May SST in 2011, and April Chl-*a* in 2014 and 2015), we used data for the 9 × 9 km cell that contains Sea Spray. Using the dates for which data were available for both cell sizes, we found high correlations for SST (*r* = 0.99 for 2005–2011 and 2012–2016) and Chl-*a* (*r* = 0.96 for 2005–2011 and *r* = 0.93 for 2012–2016) between the two cell sizes, showing the utility of 9 × 9 km cell data. For marine shores, small differences between satellite Chl-*a* data and local data from in-situ measures may exist. However, satellite data are the only Chl-*a* descriptors available for Sea Spray for the studied period. These data should be adequate for this study for at least two reasons. On the one hand, the study focuses mainly on interannual differences and such differences are relatively large (see Results), suggesting that the signal is stronger than potential noise. On the other hand, the study identifies Chl-*a* as important for barnacle larvae (see Results) and, on open shores, the pelagic larvae likely respond to Chl-*a* changes across extensive areas that may be covered well by the cells scanned by satellites.

To evaluate possible SST and Chl-*a* forcing on barnacle recruitment, we used the April and May monthly means of SST and Chl-*a* from 2005 to 2016. We used May means because recruits start to appear in early May (Ellrich, Scrosati & Molis, 2015) and April means because the nauplius larvae of *S. balanoides* develop over 5–6 weeks in coastal waters (Bousfield, 1954; Drouin, Bourget & Tremblay, 2002) before reaching the settling cyprid stage. The use of April means was further supported by recruit counts done repeatedly in 12 nearby quadrats during the 2013 recruitment season. For those quadrats, mean recruit density was 177 recruits dm^−2^ on 4 May, 417 recruits dm^−2^ on 13 May, 762 recruits dm^−2^ on 24 May, and 963 recruits dm^−2^ on 6 June. As dead recruits (indicated by empty shells on the substrate) were rare, these observations reveal that most of the new recruits appeared during May, indicating that most of the larvae that generated the June value of recruit density were in the water in April. We did not use June monthly means of SST and Chl-*a* because each year recruit density was measured before the end of June.

To evaluate if barnacle recruit density differed among years during the study period, we performed a one-way analysis of variance (Sokal & Rohlf, 2012). We evaluated the statistical influence of SST and Chl-*a* on barnacle recruit density through a model selection approach (Anderson, 2008). Considering the yearly mean of recruit density as the dependent variable, we compared the linear models representing all possible combinations of April SST, May SST, April Chl-*a*, and May Chl-*a* (15 models) based on their respective value of the corrected Akaike’s information criterion (AICc). With the 15 AICc scores, we calculated the weight of evidence for each model. Then, we assessed the plausibility of each model by calculating the corresponding evidence ratio, that is, the ratio between the weight of evidence for the best model (the one with the lowest AICc value) and that for the corresponding model (Anderson, 2008). We calculated the adjusted squared correlation coefficient (*R*^2^) for the most plausible models to determine the amount of variation in recruit density that could be explained by the corresponding combination of SST and Chl-*a*.

Given that April SST and April Chl-*a* were found relevant for barnacle recruitment (see Results), we examined factors that could explain the interannual changes in these two pelagic traits. We considered air temperature (AT) to interpret April SST changes and sea ice and winds to interpret April Chl-*a* changes. We considered AT because, on the Gulf of St. Lawrence, SST was found to lag AT by half a month (Galbraith et al., 2012). We used data from Environment Canada (2016) to calculate the month-long averages of AT for Caribou Point (45°46′N, 62°40′W, the closest weather station with AT data) centered on 31 March for the 2005–2016 period and, then, we examined the correlation with April SST. Sea ice develops extensively on the Gulf of St. Lawrence in winter and melts between late winter and early spring (Galbraith et al., 2015). We reasoned that the abundance of coastal phytoplankton at Sea Spray in April could be inversely related to the extent of ice cover, as sea ice reduces irradiance. Thus, we used daily ice charts covering Sea Spray (Canadian Ice Service, 2016) to examine correlations between April Chl-*a* for the 2005–2016 period and four measures of ice load: the Julian day when ice cover dropped below 10% for the last time every year and the mean ice cover for April and March, both separately and combined. Wind data were available for April for Caribou Point only for 2008–2016 (Environment Canada, 2016). For those years, April Chl-*a* was, on average, 63% higher along 30 km of coastline centered at Sea Spray (8.3 ± 1.4 mg m^−3^, n = 9 years; yearly April means calculated using data for nine cells along that coastal range) than along a similar length on Northumberland Strait waters 25 km offshore (5.1 ± 0.9 mg m^−3^, n = 9 years; yearly April means calculated using data for nine cells along the strait). Thus, we deemed that alongshore winds would be related to higher Chl-*a* values at Sea Spray than onshore winds. The coastline including Sea Spray is relatively linear for 30 km and it is oriented at an angle of ∼60° measured clockwise to the right of a meridian, facing Northumberland Strait waters to the northwest (Fig. 1). Thus, we classified onshore winds as those coming from a sector between 290°–10° (from offshore Northumberland Strait waters) and alongshore winds as those coming from sectors between 20°–100° and between 200°–280° (all angles measured clockwise relative to a meridian, with 0° to the north). Then, for the 2008–2016 period, we calculated the April ratio between the number of days with onshore winds and the number of days with alongshore winds and, then, we analyzed the correlation between that ratio and April Chl-*a*. We did the data analyses for this paper using JMP 9.0 for MacOS (AICc calculations) and STATISTICA 12.5 for Windows (all other analyses).

## Results

**Figure 3:**
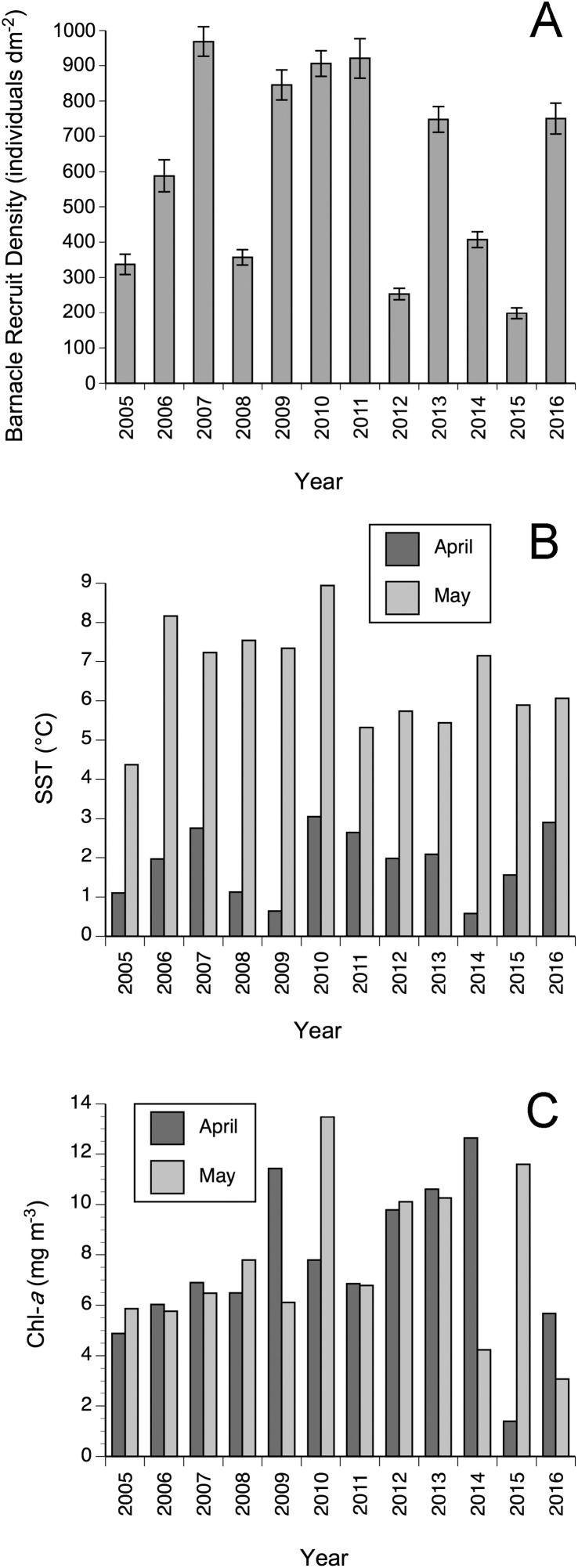
Annual changes in (A) mean (± SE) barnacle recruit density, (B) April and May SST, and (C) April and May Chl-*a* between 2005–2016.

Barnacle recruit density varied among years between 2005 and 2016 (*F*_11,363_ = 60.08, *P* < 0.0001). Yearly means ranged between 198.4 recruits dm^−2^ (in 2015) and 968.6 recruits dm^−2^ (in 2007; Fig. 3). The highest density of recruits observed in a single quadrat during the study period was 1,457 recruits dm^−2^ (in 2011) and the lowest was 34 recruits dm^−2^ (in 2015). Adult barnacles (those surviving from previous years) were always in low abundance, with an average of 9.2 individuals dm^−2^ for the study period, calculated from the 375 quadrats surveyed to measure recruit density. Other sessile species that are common in nearby wave-sheltered habitats (fucoid algae –*Fucus* spp. and *Ascophyllum nodosum*– and blue mussels –*Mytilus edulis*–) were very rare in the surveyed wave-exposed habitats in June. Small thalli of *Fucus* spp. became common in the surveyed habitats towards the fall each year, but were apparently removed by ice scour in every subsequent winter. Predatory dogwhelks (*Nucella lapillus*) and herbivorous snails (*Littorina* spp.) are also common in nearby wave-sheltered habitats, but they were also very rare in the wave-exposed habitats surveyed during the barnacle recruitment season.

**Figure 4:**
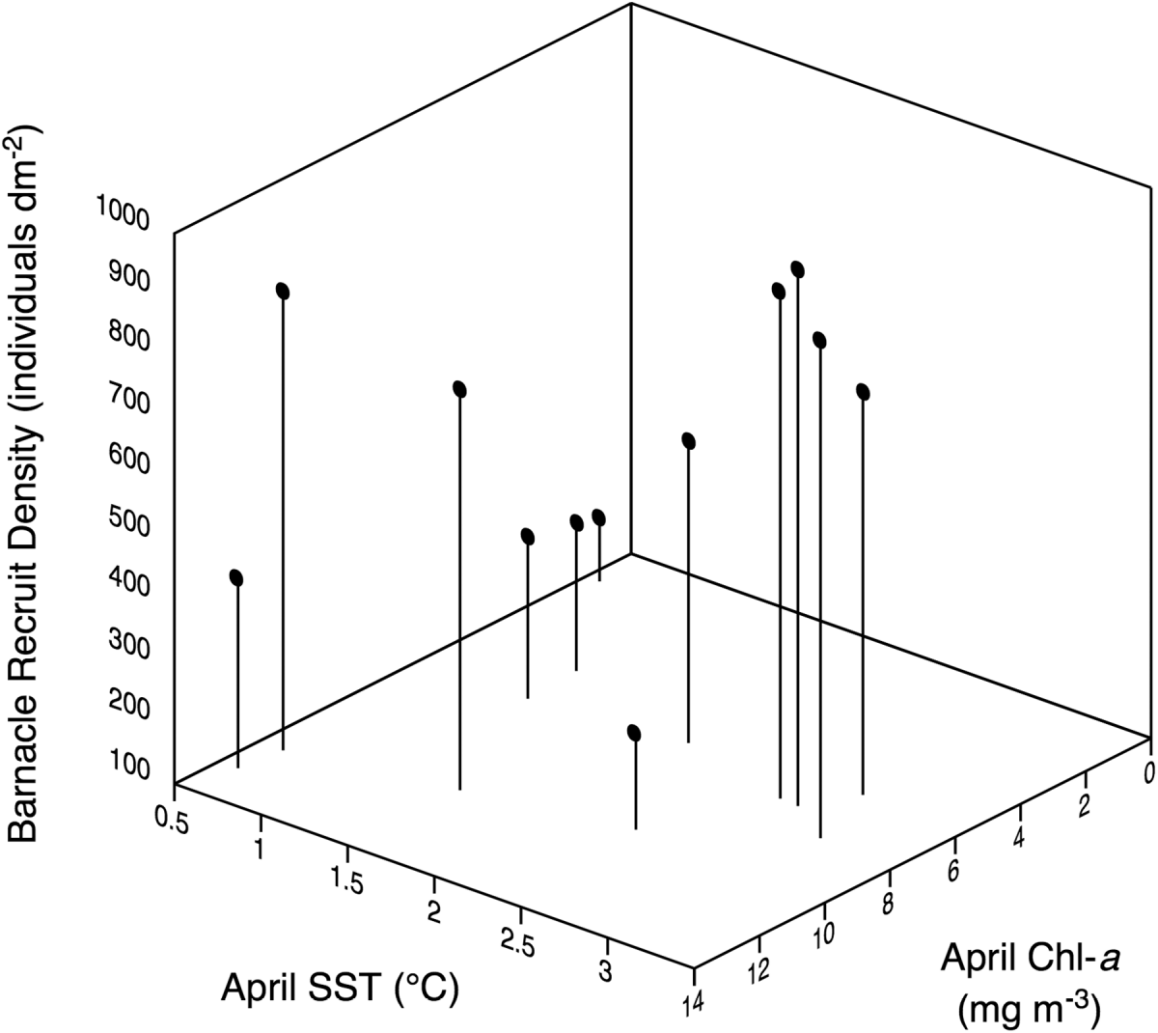
Relationship between barnacle recruit density in June, April SST, and April Chl-*a* between 2005–2016.

SST and Chl-*a* also varied between 2005 and 2016 (Fig. 3). The model comparisons based on AICc scores revealed that the best model included only April SST as independent variable (Table 2), explaining 32% (adjusted *R*^2^ = 0.32) of the interannual variation in barnacle recruit density (Table 2) through a positive relationship. This model was just 1.6 times more plausible than the next best model, which included April SST and April Chl-*a* as independent variables and explained 51% (adjusted *R*^2^ = 0.51) of the interannual variation in barnacle recruit density (Table 2). The combined influence of these two variables on recruitment was positive (Fig. 4). The other models in the set were considerably less plausible, given that their evidence ratios were higher than five (Table 2).

**Table 2:** Comparison of the 15 models representing all possible combinations of April SST, May SST, April Chl-*a*, and May Chl-*a*, considering barnacle recruit density in June as the dependent variable. The second column shows the intercept of each model (represented by each row), while columns 3–6 show the regression coefficient for each independent variable included in the corresponding model.

Independent variables in the model	Intercept	April SST	May SST	April Chl-*a*	May Chl-*a*	adj. *R*^2^	AICc	Evidence ratio
April SST	261.10	185.01	–	–	–	0.32	172.86	1
April SST, April Chl-*a*	−121.64	226.83	–	40.43	–	0.51	173.79	1.58
April SST, May SST	−87.56	177.24	55.02	–	–	0.39	176.34	5.68
May SST	172.93	–	65.70	–	–	0.10	176.34	5.69
April SST, May Chl-*a*	398.81	205.54	–	–	−23.08	0.38	176.47	6.05
April Chl-*a*	438.38	–	–	22.32	–	0.06	176.79	7.13
May Chl-*a*	672.98	–	–	–	−8.70	0.01	177.44	9.85
April SST, April Chl-*a*, May Chl-*a*	12.83	243.03	–	38.86	−20.04	0.55	178.93	20.71
April SST, May SST, April Chl-*a*	−311.28	218.01	35.44	36.74	–	0.53	179.42	26.52
May SST, April Chl-*a*	95.03	–	57.56	17.47	–	0.13	180.57	47.16
May SST, May Chl-*a*	243.47	–	70.12	–	−13.07	0.12	180.79	52.54
April SST, May SST, May Chl-*a*	22.72	199.81	62.65	–	−26.58	0.47	180.98	57.81
April Chl-*a*, May Chl-*a*	486.84	–	–	21.53	−5.57	0.06	181.46	73.55
April SST, May SST, April Chl-*a*, May Chl-*a*	−200.58	234.49	43.39	34.12	−22.84	0.59	186.66	989.31
May SST, April Chl-*a*, May Chl-*a*	158.69	–	61.89	15.64	−10.28	0.14	186.69	1,005.26

**Figure 5:**
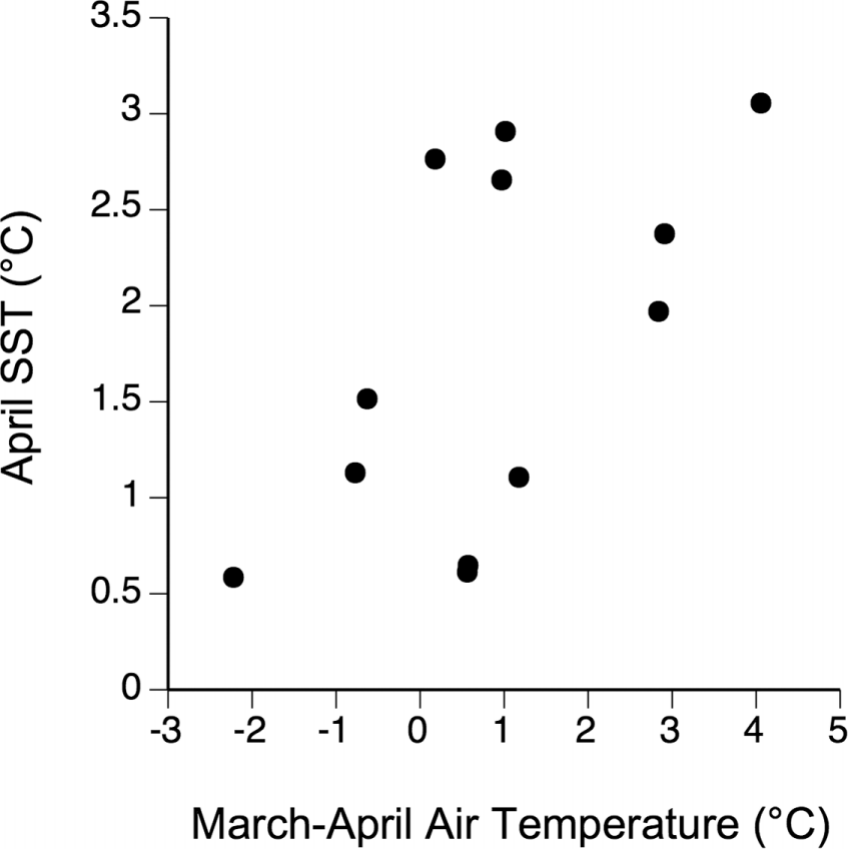
Relationship between air temperature (month-long averages centered on 31 March) and April SST between 2005–2016.

**Figure 6:**
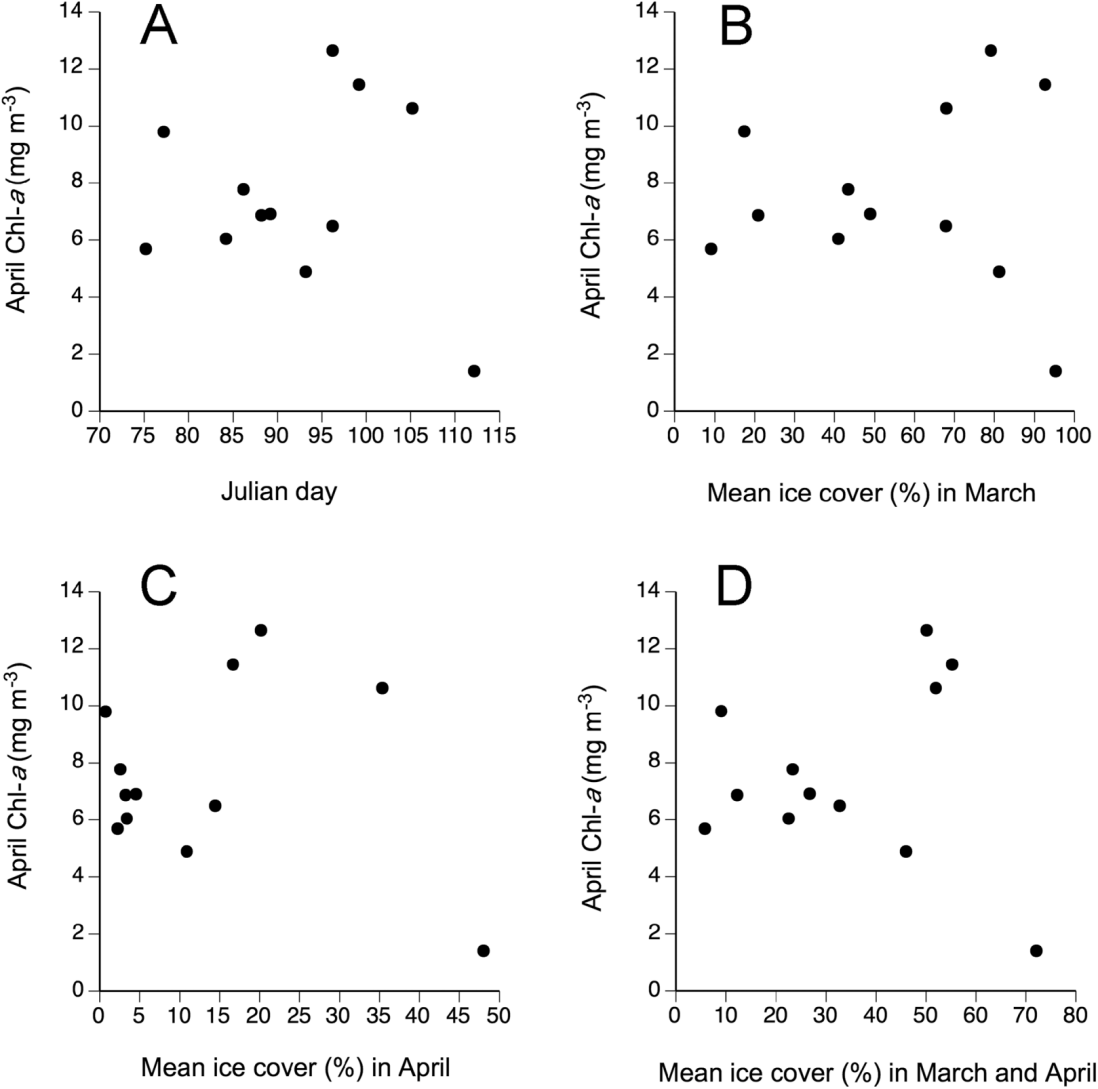
Relationship between April Chl-*a* and four measures of sea ice load: (A) Julian day when ice cover dropped below 10% for the last time every year and (B, C, and D) mean ice cover for April and March, both separately and combined.

**Figure 7:**
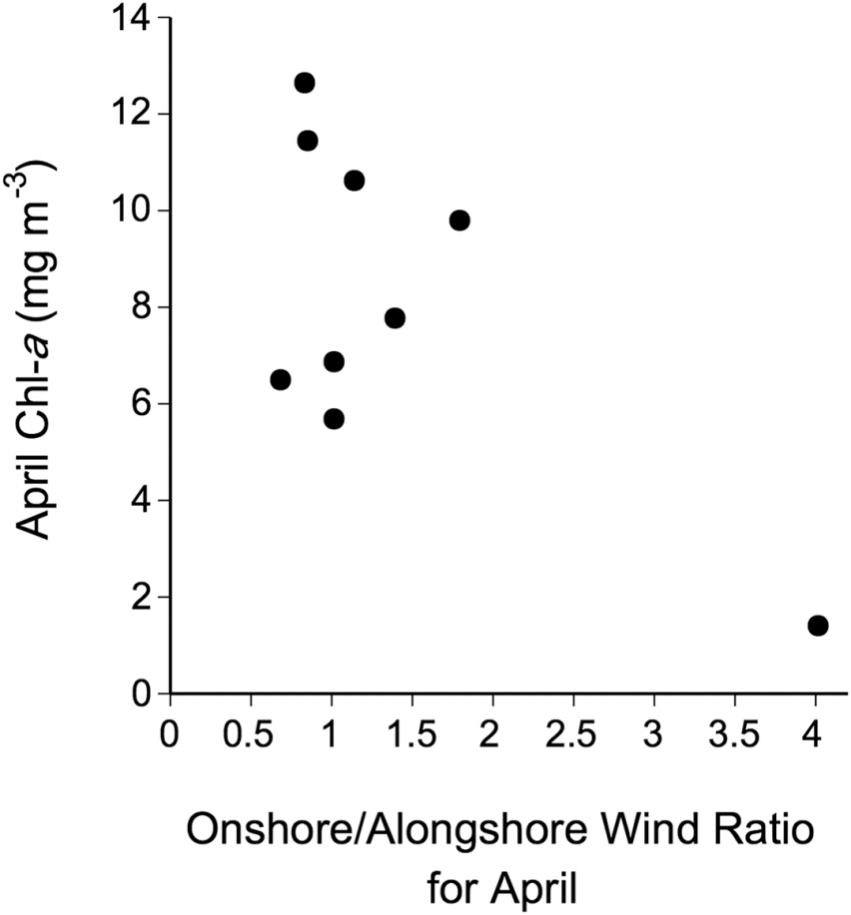
Relationship between April Chl-*a* and the April ratio between the number of days with onshore winds and the number of days with alongshore winds between 2005–2016.

Between 2005 and 2016, April SST and AT were positively correlated (*r* = 0.59, *P* = 0.042; Fig. 5). Linear correlations between April Chl-*a* and the four tested measures of ice load during this period were nonsignificant (*P* values between 0.55–0.95) and no nonlinear relationship was apparent either (Fig. 6). The April ratio between the number of days with onshore winds and the number of days with alongshore winds was negatively related to April Chl-*a* (*r* = −0.68, *P* = 0.045; Fig. 7).

## Discussion

This study reveals that intertidal barnacle recruitment has consistently occurred during the last 12 years at Sea Spray, our long-term reference location in Atlantic Canada. Adult barnacles are rare in the spring in wave-exposed habitats on this coast, mostly as a result of winter ice scour, as adult barnacle density in the fall is higher once the recruits from the preceding spring have grown (Belt, Cole & Scrosati, 2009). In the spring, adult barnacles are usually more abundant in wave-sheltered habitats (Belt, Cole & Scrosati, 2009), where winter ice scour is less intense (Scrosati & Heaven, 2006). Thus, the pool of larvae that repopulates wave-exposed habitats so abundantly in the spring likely comes from exposed and sheltered habitats hosting reproductive barnacles (MacPherson, Scrosati & Chareka, 2008). Identifying spatial sources of larvae could be investigated by looking at larval dispersal and spatial genetics in relation to local reproductive output and water movement (Caley et al., 1996; Jonsson, Berntsson & Larsson, 2004; Selkoe et al., 2016).

Although recruitment occurred every spring at Sea Spray, the intensity varied across years. Our model selection approach identified April SST as the best explanatory variable for recruitment, although the model with April SST and April Chl-*a* was similarly important, remarkably explaining half of the interannual variation in recruitment. The other tested models were considerably less plausible, according to model selection rules (Anderson, 2008). The first barnacle recruits appear on the studied coast in early May (Ellrich, Scrosati & Molis, 2015). Our observations during the 2013 recruitment season indicated that most of the recruits composing the June recruit count appeared on the shore during May, and the phytoplanktotrophic larvae of *S. balanoides* go through nauplius stages for 5–6 weeks before reaching the settling stage (Bousfield, 1954; Drouin, Bourget & Tremblay, 2002). Therefore, the statistical relevance of April SST and April Chl-*a* suggests that water temperature and pelagic food supply influence intertidal recruitment primarily through a positive influence on pelagic larvae. Interestingly, Barnes (1956) found that the development of *S. balanoides* larvae on a UK shore improved with planktonic diatom abundance.

The present study evaluates multiannual patterns in barnacle recruitment at one NW Atlantic location. Data obtained for single years at other NW Atlantic locations further support the notion that Chl-*a* is important for recruitment in this region. Those studies also evaluated *S. balanoides* recruitment at an elevation of 2/3 of the intertidal range in wave-exposed habitats. In 2007 on the Damariscotta area in Maine (USA), recruit density was similar to that found at Sea Spray in the same year (Fig. 3), in agreement with the similarity in Chl-*a* found for both shores (Cole et al., 2011). Where Chl-*a* was three times lower, such as the west coast of Cape Breton Island, in northern Nova Scotia, recruit density in 2007 was considerably lower (Cole et al., 2011). In 2013 on Deming Island, near Whitehead on the Atlantic coast of Nova Scotia, recruit density was ∼500 recruits dm^−2^ (Ellrich & Scrosati, 2016) and April Chl-*a* was ∼10 mg m^−3^ (National Aeronautics and Space Administration, 2016), similar to what we found at Sea Spray in that year (Fig. 3). In 2014, Deming Island exhibited a higher Chl-*a* than a location farther south on the Atlantic coast (Tor Bay Provincial Park) and a higher recruit density as well (Petzold & Scrosati, 2014).

Regarding SST, other studies have also noted its importance for the recruitment of *S. balanoides*. For example, on the Atlantic coast of England and France, recruitment was higher after the cold winters of 2010 and 2011 than after the warmer winter of 2012 (Abernot-Le Gac et al., 2013; Rognstad, Wethey & Hilbish, 2014). That coast exhibits a winter SST range of 7–13 °C and a laboratory experiment found that the survival of *S. balanoides* embryos decreases from 7 to 13 °C (Rognstad & Hilbish, 2014), potentially explaining their negative SST–recruitment relationship. However, with the low SST range experienced on the Sea Spray coast early every year (below 3 °C in April; Fig. 3), spring recruitment was actually positively related to April SST. Thus, together, these studies suggest that a unimodal relationship between SST early in the year and barnacle recruitment in the spring might exist for *S. balanoides*. At the low SST range that characterizes the Sea Spray coast early in the year, increasing SST might enhance embryo and larval survival. Future research could address these possibilities (past research on SST and larval survival did not consider low enough SST values; Harms, 1984).

Studies on *S. balanoides* recruitment on the NE Atlantic coast have found similar (Hawkins & Hartnoll, 1982; Kendall et al., 1985; Jenkins et al., 2000; Kent, Hawkins & Doncaster, 2003; Rognstad, Wethey & Hilbish, 2014) and higher (Kendall et al., 1985; Jenkins et al., 2000; Jenkins, Murua & Burrows, 2008) values than our study. In combination, those studies sampled a higher diversity of conditions (wave exposure, elevation, and food supply) than ours, which may explain their higher range of recruitment (see also below in this paragraph). On the NE Pacific coast, intertidal barnacle recruitment is often high. For example, on the coasts of Oregon and northern California (USA), recruits of two barnacle species (*Balanus glandula* and *Chthamalus dalli*) appear throughout most of the year and, considering both species together, can reach mean densities of ∼1800 recruits dm^−2^ in just one month (Navarrete, Broitman & Menge, 2008). That coast is characterized by upwelling. In many places, the frequent alternation of upwelling with relaxation periods allows for barnacle larvae to remain near the coast (persistent upwelling takes larvae offshore) and favours high levels of nearshore Chl-*a* (above 23 mg m^−3^), ultimately stimulating intertidal barnacle recruitment (Menge & Menge, 2013). The low SST values shortly after ice melt at Sea Spray in April probably further contribute to the lower recruit densities often observed at Sea Spray relative to those coasts. On the other hand, the higher recruitment rates reported for the NE Atlantic (Jenkins et al., 2000) and NE Pacific (Navarrete, Broitman & Menge, 2008) coasts may respond in part to the relative elevation where recruitment was measured. While our study surveyed the lower part of the upper third of the intertidal range, the other studies surveyed middle (Jenkins et al., 2000) and middle-to-low (Navarrete, Broitman & Menge, 2008) elevations. In 2006, we measured barnacle recruitment at the low, middle, and high intertidal zones of wave-exposed habitats at Sea Spray. Mean recruit density was three times higher at the middle zone and two times higher at the low zone than at the high zone (MacPherson & Scrosati, 2008). This suggests that recruitment differences between these coasts, although present, could be smaller if data were available for the same relative elevation.

As April SST and April Chl-*a* contributed to explain the interannual changes in barnacle recruitment at Sea Spray, we examined factors potentially explaining SST and Chl-*a* changes. The positive AT–SST association suggests that climatic phenomena driving AT in late winter and early spring may indirectly influence recruitment through effects on April SST. The measures of sea ice load that we examined, however, did not explain Chl-*a* changes, so it is not evident whether sea ice has any influence. Lastly, we found evidence that wind direction influences Chl-*a* at Sea Spray. Our results suggest that onshore winds would take phytoplankton-limited surface waters from the central Northumberland Strait to the coast, while alonghore winds would help to retain phytoplankton near the coast, indirectly favouring barnacle recruitment.

Overall, this appears to be the first study monitoring invertebrate recruitment from NW Atlantic rocky intertidal habitats for more than a decade. The continuation of this project over the years should consolidate our understanding of recruitment fluctuations and the role of seawater temperature and planktonic food supply. Surveying more locations would provide the spatial dimension that single-location data cannot do by design. To this aim, in 2014 we began monitoring barnacle recruitment in wave-exposed locations along the Atlantic coast of Nova Scotia (Scrosati & Petzold, 2016), but it is early to identify interannual trends in those places. Continued surveys in Atlantic Canada should enrich benthic–pelagic coupling theory, as shown by years of recruitment studies on other coasts (Navarrete, Broitman & Menge, 2008; Menge & Menge, 2013; Menge et al., 2015). Ultimately, such datasets should facilitate the prediction of ecological responses to environmental changes (Mieszkowska et al., 2014; Schiel et al., 2016).

## Supplemental Information

10.7717/peerj.2623/supp-1 Supplemental Information 1 Full dataset used to make the calculations described in the paper.

## Additional Information and Declarations

### Data Deposition

The following information was supplied regarding data availability: The full dataset is available through the corresponding PeerJ Preprints article: Scrosati RA, Ellrich JA. 2016. Intertidal barnacle recruitment in Nova Scotia (Canada) between 2005–2016: relationships with sea surface temperature and phytoplankton abundance. PeerJ Preprints 4:e2355v1 DOI 10.7287/peerj.preprints.2355v1.
